# Difficult Laparoscopic Cholecystectomy and Trainees: Predictors and Results in an Academic Teaching Hospital

**DOI:** 10.1155/2017/6467814

**Published:** 2017-06-05

**Authors:** Hussein M. Atta, Ashraf A. Mohamed, Alaa M. Sewefy, Abdel-Fatah S. Abdel-Fatah, Mohammed M. Mohammed, Ahmed M. Atiya

**Affiliations:** Department of General Surgery, Faculty of Medicine, Minia University, El-Minia 61519, Egypt

## Abstract

Laparoscopic cholecystectomy (LC) is one of the first laparoscopic procedures performed by surgical trainees. This study aims to determine preoperative and/or intraoperative predictors of difficult LC and to compare complications of LC performed by trainees with that performed by trained surgeons. A cohort of 180 consecutive patients with cholelithiasis who underwent LC was analyzed. We used univariate and binary logistic regression analyses to predict factors associated with difficult LC. We compared the rate of complications of LCs performed by trainees and that performed by trained surgeons using Pearson's chi-square test. Patients with impacted stone in the neck of the gallbladder (GB) (OR, 5.0; 95% CI, 1.59–15.77), with adhesions in the Triangle of Calot (OR, 2.9; 95% CI, 1.27–6.83), or with GB rupture (OR, 3.4; 95% CI, 1.02–11.41) were more likely to experience difficult LC. There was no difference between trainees and trained surgeons in the rate of cystic artery injury (*p* = .144) or GB rupture (*p* = .097). However, operative time of LCs performed by trained surgeons was significantly shorter (median, 45 min; IQR, 30–70 min) compared with the surgical trainees' operative time (60 min; IQR, 50–90 min). Surgical trainees can perform difficult LC safely under supervision with no increase in complications albeit with mild increase in operative time.

## 1. Introduction

Laparoscopic cholecystectomy (LC) is the standard of care for patients with cholelithiasis. Several randomized controlled trials and systematic reviews have demonstrated the effectiveness and safety of LC for the treatment of symptomatic cholelithiasis [1–4]. The rapid acceptance of LC as the standard of care for patients with gallstones has been attributed to several benefits including decreased patient morbidity, faster recovery, and shorter hospital stay when compared to open cholecystectomy [5–7].

LC is one of the first laparoscopic procedures performed by surgical trainees. Despite the establishment of formal training in laparoscopic surgery and the improvement in laparoscopic technology, still, there is a perception that performance of LCs in teaching hospitals with continuous inflow of trainees may be attended with difficult LC, increased conversion, and complication rates [8]. While several studies have reported a variable assembly of different preoperative and operative risk factors associated with difficult LC and conversion to open cholecystectomy [9–13], the performance of surgical trainees with different training backgrounds has not been adequately addressed.

Although conversion of LC to open cholecystectomy is considered an important outcome of LC, however, currently, conversion rate is less common (2.6%–5.2%) than other surrogate parameters of difficult LC such as operative time more than 60 min, adhesions in the Triangle of Calot, cystic artery injury, or spillage of stones [14–16].

This study is conducted to determine predictors of difficult LC, defined as operative time more than 60 min and/or cystic artery injury, in the setting of a single academic teaching hospital and in particular to compare the outcomes of LCs performed by surgical trainees with those performed by trained surgeons.

## 2. Methods

This retrospective cohort included 180 consecutive patients with cholelithiasis who underwent LC at Minia University Hospital, El-Minia, Egypt, from November 2014 to October 2016. The study protocol was approved by the Faculty of Medicine Minia University Council. Informed consent was obtained from all patients, and data were collected prospectively. In order to have a homogenous patient population, we excluded patients with acute cholecystitis, pancreatitis, common bile duct (CBD) stone, and those who underwent combined LC with any other laparoscopic interventions including laparoscopic CBD exploration. All LCs were performed on an elective basis. LCs were performed by surgeons with three years of general surgery training and are referred to as surgical trainees or by experienced laparoscopic surgeons who had more than five years of surgery training and are referred to as trained surgeons. During their training, surgical trainees assisted in at least 150 LCs but did not assume the role of surgeon, while trained surgeons had performed more than 25 unsupervised LCs [17].

Both groups were assigned to LCs according to their duty schedule; thereby, no surgeon selection was attempted. However, surgical trainees were supervised in the theater by a nonscrub trained surgeon. All LC procedures were completed by the initial operating surgeon. Thus, this study setting reflects a real setting of a midsize university teaching hospital. LCs were performed either with the retrograde approach (dissection initiated from the Triangle of Calot upward to the fundus of the gallbladder) or with the dome-down technique (removing the gallbladder from the gallbladder bed first) according to the surgeon discretion in lieu of the severity of adhesions at the Triangle of Calot. Retrograde approach was used when there are minimal, easily dissectible adhesions, while dome-down technique was used in the presence of severe adhesions. Difficult LCs were defined as LC with operative time of more than 60 min, or with injury to the cystic artery before ligation or clipping [16]. Patients' characteristics including demographic, clinical, ultrasonographic, and operative parameters that could contribute to predicting operative difficulties were analyzed.

### 2.1. Statistical Analysis

Categorical variables are presented as counts and percentages. Continuous variables are presented as mean ± standard deviation or median (25th–75th interquartile range, IQR) for normally or not normally distributed variables, respectively. Shapiro-Wilk test was used to test for a normal distribution. Univariate analysis of patients' characteristics was performed to identify variables associated with difficult LC. Categorical variables were compared using Fisher exact test, and continuous variables not normally distributed were compared using the nonparametric Mann-Whitney *U* test. To identify independent predictors of difficult LC, variables with a *p* value <0.05 were subsequently entered into a binary logistic regression model [18]. Validity of the model was checked using the Hosmer and Lemeshow goodness of fit test [19]. The difference between the rate of complications of LCs performed by surgical trainees and that performed by trained surgeons was compared using Pearson's chi-square or Mann-Whitney *U* test. For all statistical analyses, two-tailed tests were used. Statistical analysis was performed using the software Statistical Package for Social Sciences, SPSS version 13 (SPSS, Chicago, IL, USA). A *p* value of <0.05 was considered statistically significant.

## 3. Results

### 3.1. Patient Characteristics

A total of 180 consecutive patients underwent LC at Minia University Teaching Hospital from November 2014 to October 2016. Fifty eight LCs (32%) fulfilled the criteria of difficult LC defined as operative time of more than 60 min or injury to the cystic artery. Coronary heart disease, hemolytic anemia, and hepatitis C virus infection each occurred in a single patient and were not entered in the analysis. Twenty three per cent of LCs was performed by surgical trainees. Cystic artery injury occurred in six LCs, and there was no CBD injury. A single LC was converted to open cholecystectomy in this cohort due to inability to control bleeding from injured cystic artery.

#### 3.1.1. Risk Factors for Difficult LC

Comparison of patient characteristics between difficult and easy LCs identified nine risk factors for difficult LC that differed significantly (Table 1). Injury of the cystic artery and its related blood loss > 50 mL and operative time ≥ 60 min were not included in the regression analysis because they constitute the definition of difficult LC. Identified risk factors include, male gender, gallbladder (GB) wall thickness ≥ 4 mm, GB fluid containing sludge, impacted stone in the neck of the GB, pericholecystic fluid collection, adhesions in the Triangle of Calot, ruptured GB, spilled stones, and surgeon skill of less than ten LCs.

#### 3.1.2. Regression Model Performance

A binary logistic regression analysis was performed to determine the effects of risk factors on the likelihood that patients having difficult LC. The binary logistic regression model was statistically significant, *χ*^2^ = 67.202, *p* < .001. The Hosmer and Lemeshow goodness of fit test suggests that the model is a good fit to the data as *p* = 0.460 is nonsignificant [19]. The model explained 43.5% (Nagelkerke *R*^2^) of the variance in difficult LC. The classification table (Table 2) is a method to evaluate the predictive accuracy of the logistic regression model. In this table, the observed values for the dependent outcome and the predicted values (at a cutoff value of *p* = 0.50) are cross-classified. Our model correctly predicts 81.7% of cases. We calculated the error rates from the classification table output. A false positive would be predicting that difficult LC would occur when, in fact, it did not. Our model predicted difficult LC 43 times. That prediction was wrong nine times, for a false positive rate of 9/43 = 20.9%. A false negative would be predicting that difficult LC would not occur when, in fact, it did occur. Our model predicted difficult LC not to occur in 137 times. That prediction was wrong 24 times, for a false negative rate of 24/137 = 17.5%.

### 3.2. Predictors of Difficult LC

This model suggests that impacted stone in the neck of the GB, adhesions in the Triangle of Calot, and GB rupture during LC are independent predictors of difficult LC (Table 3). Patients with impacted stone in the neck of the GB are about five times (odds ratio [OR], 5.0; 95% confidence interval [CI], 1.59–15.77) likely to undergo a difficult LC. This model also shows that patients with adhesions in the Triangle of Calot (OR, 2.9; 95% CI, 1.27–6.83) or with GB rupture during LC (OR, 3.4; 95% CI, 1.02–11.41) are about three times more likely to experience difficult LC (Table 3).

### 3.3. Outcome of Trainee-Performed LCs

**Figure 1:**
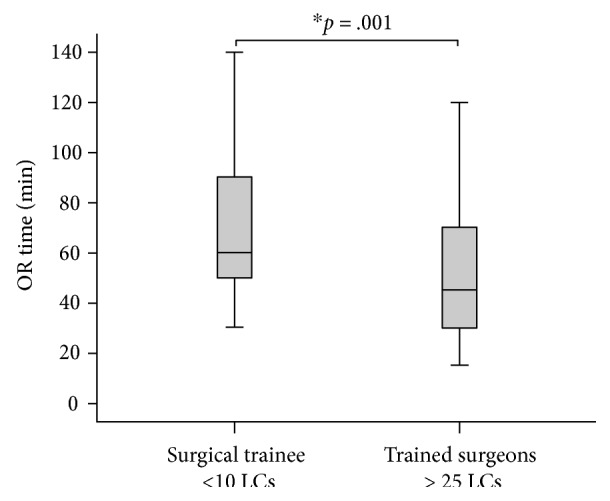
Boxplots (median, interquartile range, max, and min) of LC operative time of surgical trainees and trained surgeons (min). ∗significant differences between surgical trainees and trained surgeons.

Although our regression model did not select trainees as a predictor of difficult LC, however, we hypothesized that there may be a difference between the rate of complications of LCs performed by trainees with experience of less than ten LCs and that performed by trained surgeons with skills of more than 25 unsupervised LCs. We found that there is no statistically significant difference between trainees and trained surgeons in the rate of cystic artery injury (4.9% and 1.0%, Pearson's chi-square, *p* = 0.144) or GB rupture (17.1% and 30.7%, *p* = 0.097). As expected, we found that operative time of LCs performed by trained surgeons was significantly shorter (median, 45 min; IQR, 30–70 min) compared with surgical trainees' operative time (60 min; IQR, 50–90 min) (Mann-Whitney *U* test, *p* = 0.001) (Figure 1).

## 4. Discussion

This study suggested that impacted stone in the neck of the GB, the presence of adhesions in the Triangle of Calot, GB rupture, and injury to the cystic artery predicted increase in the likelihood of having difficult LC. Furthermore, we showed also that in case of difficult LC performed by surgical trainees under direct supervision of trained surgeons, there was no increase in the LC complications, cystic artery injury, GB rupture, or conversion when compared with trained surgeons. There is, however, infrequent increase in the operative time of LCs performed by surgical trainees.

Currently, LC is the standard of care for patients with cholelithiasis and is the first laparoscopic surgical procedure to be performed by general surgery trainees in many teaching hospitals [20]. These laparoscopic skills must be passed on to junior surgeons without compromising patient safety. In our surgical training program, we do not use surgical simulators or cadaveric surgery for laparoscopic surgery training but we solely rely on extended operative assistance. Our surgical trainees start performing LC only after assisting in at least 150 LCs during their previous three years of surgical training. This study showed also that surgical trainees, who performed LCs under direct supervision of trained surgeons, had no increase in the LC complications when compared with trained surgeons. However, the operative time is longer in LCs performed by surgical trainees compared with trained surgeons. In agreement with our results, Lavy et al. reported a comparative study of LC performed by residents with that performed by senior surgeons [20]. They found that the only significant difference between the groups was a longer operative time, while the conversion rate and complication rate were the same. In a similar study comparing consultant surgeons, trainee surgeons, and trained surgeons, the authors found that there were no differences among the three groups in conversion rates, bile duct injury rates, general complication rates, or length of stay; however, the duration of operation in the trainee surgeons was significantly longer compared to the other two groups [21]. In the setting of LC for acute cholecystitis, Abelson et al. reported that advanced laparoscopic fellowship-trained surgeons had significantly lower conversion rate and shorter operative time than the nonfellowship-trained surgeons; however, the complication rates were not significantly different [22].

The low incidence of conversions in our cohort of 180 consecutive patients with gall stone disease is primarily due to the fact that this series did not include LC performed in patients with acute cholecystitis, pancreatitis, or CBD stone. A study from a single university medical center reported a conversion rate of 2.6%, and the diagnosis of acute cholecystitis was more common among converted cases [15]. In a recent analysis of preoperative risk factors for conversion from a prospective U.K. database of 8820 patients, Sutcliffe et al. reported a rate of conversion when the indication for LC was for cholecystitis (6.5%) to be higher than that for colic (1.2%) or for pancreatitis (2.1%) but only lower than that of CBD stone (9.1%) [23].

## 5. Conclusion

This study demonstrated that operative complications of LC performed by surgical trainees who had extended operative exposure and who performed LC under direct supervision of trained surgeons are not different from those performed by trained surgeons except in moderate increase of operative time.

## Figures and Tables

**Table 1:** Comparison of patient characteristics between difficult and easy LCs.

Characteristics	Difficult LC (*n* = 58)	Easy LC (*n* = 122)	*p* value
*Preoperative characteristics*			
Male gender^†^	16 (27.6%)	17 (13.9%)	0.038
Age >65 years	1 (1.7%)	4 (3.3%)	1.000
BMI, ≥30 kg/m^2^	10 (17.2%)	19 (15.5%)	0.829
Smoking	6 (8.6%)	5 (4.1%)	0.179
Elevated liver enzymes	1 (1.7%)	1 (0.8%)	0.542
Previous abdominal operation	12 (20.1%)	21 (17.2%)	0.681
Hypertension	5 (8.6%)	8 (6.6%)	0.412
Diabetes mellitus	3 (5.2%)	2 (1.6%)	0.330
Liver cirrhosis	3 (5.2%)	2 (1.6%)	0.330
Previous biliary hospitalization	13 (22.4%)	18 (14.8%)	0.212
Palpable GB	0	3 (2.5%)	0.552
GB wall thickness, ≥4 mm^†^	38 (65.5%)	49 (40.2%)	0.002
GB transverse diameter, <2, >5 cm	18 (31.0%)	40 (32.8%)	0.866
GB sludge^†^	32 (55.2%)	22 (18%)	0.000
Impacted stone in the neck of GB^†^	23 (39.7%)	7 (5.7%)	0.000
Pericholecystic fluid collection^†^	5 (8.6%)	1 (0.82%)	0.014
CBD diameter, >10 mm	2 (3.4%)	4 (3.3%)	1.000
CBD stones	1 (1.7%)	1 (0.82%)	0.542
Surgeon LC skill, <10 LCs^†^	19 (32.8%)	22 (18%)	0.036
Surgeon LC skill, >25 LCs	26 (44.8%)	75 (61.5%)	0.028
*Intraoperative characteristics*			
Operative time, >60 min	57 (98.3%)	40 (32.8%)	0.000
Operative blood loss, >50 mL	30 (51.7%)	29 (23.8%)	0.000
Triangle of Calot adhesions^†^	36 (62.1%)	29 (23.7%)	0.000
Ruptured GB^†^	29 (50%)	19 (15.6%)	0.000
Spilled stones^†^	20 (34.5%)	10 (8.2%)	0.000
Cystic artery injury	6 (10.3%)	0	0.001
CBD injury	0	0	0
Conversion	1 (1.7%)	0	0.322

^†^characteristics included in binary logistic regression analysis.

**Table 2:** Classification table.

Observed	Predicted		
	Difficult LC		Percentage correct
	Easy	Difficult	
Easy	113	9	92.6
Difficult	24	34	58.6
Overall percentage			81.7

**Table 3:** Binary logistic regressions analysis of risk factors for difficult LC.

	Regression coefficient	Wald statistic	*p* value	Odds ratio	95% C.I.	
Impacted stone	1.614	7.628	.006	5.021	1.598	15.779
Calot's adhesions	1.079	6.305	.012	2.943	1.267	6.834
GB rupture	1.225	3.943	.047	3.405	1.016	11.413
